# Dual Imaging Biomarkers for MASLD Assessment in Bariatric Patients After Sleeve Gastrectomy

**DOI:** 10.3390/diagnostics16142246

**Published:** 2026-07-18

**Authors:** Camelia Croitoru (Oprea), Vlad-Teodor Enciu, Cătălin Copăescu, Carmen Fierbințeanu-Braticevici

**Affiliations:** 1 Ponderas Academic Hospital, 014142 Bucharest, Romania; camelia.oprea@drd.umfcd.ro (C.C.); catalincopaescu@gmail.com (C.C.); 2 Carol Davila University of Medicine and Pharmacy, 050474 Bucharest, Romania; cfierbinteanu@yahoo.com

**Keywords:** ultrasound-derived fat fraction, visceral adipose tissue, DXA, bariatric surgery, hepatic steatosis, MASLD, metabolic surgery, body composition

## Abstract

**Background and Aims:** Metabolic dysfunction-associated steatotic liver disease (MASLD) is the most prevalent chronic liver disease and frequently improves after bariatric surgery. Conventional postoperative follow-up is mainly based on weight-loss outcomes, such as Body Mass Index (BMI), excess weight loss (EWL), total weight loss (TWL), and routine biochemical parameters, which may not fully characterize hepatic fat response. Ultrasound-derived fat fraction (UDFF) provides a quantitative non-invasive assessment of hepatic steatosis, while DXA-derived visceral adipose tissue (VAT) reflects central adiposity and metabolic risk. This study aimed to evaluate whether combined UDFF and DXA-VAT assessment provides complementary information to conventional bariatric follow-up parameters after bariatric surgery. **Methods:** We conducted a prospective longitudinal cohort study on 41 patients with severe obesity who underwent bariatric surgery between July 2025 and May 2026. Anthropometric, body composition, metabolic, hepatic, and biochemical parameters were assessed at baseline (T0) and 6 months postoperatively (T1). UDFF, utilizing a DAX ultrasound transducer, was used for hepatic steatosis assessment, while DXA was performed for VAT, android fat mass, total body fat percentage, and lean mass quantification. Correlation analyses, multivariable linear regression, and an exploratory clinical discordance analysis were performed. **Results:** At 6 months after surgery, patients showed significant improvements in body weight, visceral adiposity, insulin resistance, biochemical parameters, and hepatic steatosis. VAT reduction showed the strongest association with UDFF reduction, whereas conventional weight-loss outcomes, anthropometric, and metabolic parameters did not fully identify patients with residual steatosis. These findings suggest that hepatic fat improvement after bariatric surgery is more closely related to changes in visceral adiposity than to weight loss alone. **Conclusions:** Combined UDFF and DXA-derived VAT assessment may provide a practical dual-compartment imaging framework for postoperative MASLD monitoring. This approach captures both hepatic fat response and visceral adiposity remodeling, offering information that complements conventional bariatric follow-up based on weight-loss outcomes.

## 1. Introduction

Bariatric surgery is currently the most effective therapeutic intervention for severe obesity, with accepted indications including BMI ≥ 35 kg/m^2^, regardless of the presence of obesity-related comorbidities, and BMI 30–34.9 kg/m^2^ in selected patients with type 2 diabetes or other obesity-related conditions not adequately controlled by non-surgical treatment. Beyond weight loss, bariatric surgery induces major improvements in insulin resistance, visceral adiposity, and hepatic steatosis [1]. However, postoperative follow-up is still largely centered on anthropometric and metabolic endpoints, whereas quantitative assessment of MASLD is not consistently incorporated into standardized bariatric monitoring pathways [2].

This represents an important clinical gap. Therefore, accessible and reproducible imaging-based tools are needed to monitor liver fat changes in parallel with changes in visceral adiposity and global body composition. Ultrasound-derived fat fraction (UDFF) has recently emerged as a quantitative, non-invasive ultrasound technique for assessing hepatic steatosis, with growing evidence supporting its agreement with MRI-PDFF and CAP measurements [3,4,5].

At the same time, dual-energy X-ray absorptiometry (DXA) allows objective quantification of visceral adipose tissue (VAT), a metabolically active fat compartment closely linked to insulin resistance and MASLD pathophysiology [6]. Although UDFF and DXA-derived VAT may provide complementary information, the relationship between hepatic fat reduction and visceral adiposity reduction after bariatric surgery remains insufficiently characterized. The UDFF–VAT(DXA) dual-compartment imaging approach may help integrate hepatic and body-composition monitoring into a more clinically meaningful postoperative assessment model.

Therefore, the present study aimed to evaluate changes in UDFF and DXA-measured VAT 6 months after bariatric surgery and to assess whether these imaging-based parameters provide complementary information to conventional postoperative follow-up markers, including BMI, EWL, TWL, liver enzymes, and metabolic parameters. By focusing on both hepatic fat and visceral adiposity, this study explores a combined imaging-monitoring approach that may improve the characterization of metabolic and hepatic response after bariatric surgery.

## 2. Materials and Methods

### 2.1. Design and Population

This was a prospective, single-center cohort study conducted between July 2025 and May 2026. Patients were enrolled at baseline between July 2025 and November 2025, and the 6-month postoperative follow-up assessments were completed by May 2026. The study included 41 consecutive adult patients with severe obesity who underwent laparoscopic sleeve gastrectomy after approval by the multidisciplinary bariatric committee. None of the included patients had diabetes mellitus, and no patient was receiving antidiabetic medication. Arterial hypertension, when present, was clinically controlled with standard antihypertensive therapy, mainly angiotensin-converting enzyme inhibitors or angiotensin receptor blockers, while patients with hypercholesterolemia were treated with lipid-lowering therapy, primarily statins. Detailed patient-level data regarding the number of patients receiving each treatment class, treatment duration, and postoperative treatment changes were not systematically collected. The study was approved by the Institutional Ethics Committee of Ponderas Academic Hospital (no. 378/9 July 2025), and all participants signed the informed consent.

### 2.2. Eligibility Criteria

Patients were eligible for inclusion if they were aged between 18 and 70 years, fulfilled institutional criteria for bariatric surgery in accordance with the eligibility criteria outlined in the 2022 ASMBS/IFSO guidelines, and were accepted for laparoscopic sleeve gastrectomy following multidisciplinary evaluation [7].

The main exclusion criteria were alcohol intake above the accepted threshold for MASLD diagnosis, use of medications known to induce hepatic steatosis, known chronic liver diseases other than MASLD, active cholestasis, clinically significant cardiac congestion, or incomplete baseline or 6-month follow-up data.

### 2.3. Clinical and Anthropometric Assessment

Evaluations were performed at two time points: preoperative (T0, approximately 10 days before the intervention) and at 6 months postoperative (T1).

Anthropometric measurements were performed according to standardized protocols recommended by the World Health Organization (WHO). Body weight was measured using a calibrated scale with participants wearing light clothing and no shoes. Height was measured using a stadiometer with participants standing upright in the Frankfurt plane. Waist circumference was measured using a non-elastic measuring tape at the midpoint between the lowest rib and the iliac crest at the end of a normal expiration. The following anthropometric parameters were recorded: body weight, height, BMI, and waist circumference.

### 2.4. Body Composition Assessment by DXA

Body composition was evaluated using (DXA with a GE Lunar iDXA system (GE Healthcare, Madison, WI, USA), a validated method for the assessment of total and regional body composition. Parameters analyzed included total fat mass, lean mass, VAT, and the android-to-gynoid fat ratio. All measurements were performed in accordance with standardized acquisition protocols (in the fasting state, barefoot, and wearing light clothing without metal accessories) with routine calibration and internal quality control procedures implemented to ensure precision and reproducibility.

### 2.5. Ultrasound-Derived Fat Fraction Assessment

Hepatic steatosis was quantified using UDFF measured with the Siemens ACUSON Sequoia ultrasound system equipped with the DAX transducer (Siemens Medical Solutions USA, Inc., Issaquah, WA, USA). DAX refers to the Deep Abdominal Transducer, designed for abdominal imaging in patients with increased body habitus (https://www.siemens-healthineers.com/en-us/ultrasound/dax-1, accessed on 16 July 2026).

UDFF measurements were performed in the absence of cholestasis or clinical signs of hepatic congestion. Patients were instructed to fast for at least 4 h before the examination and to rest for at least 10 min before image acquisition. Examinations were performed with the patient in the supine or slight left lateral decubitus position, with the right arm elevated above the head.

The transducer was positioned perpendicular to the liver capsule. The region of interest was placed parallel to the liver capsule, approximately 15–20 mm below the liver surface, avoiding large vessels, bile ducts, focal lesions, and artifacts. Measurements were obtained during a neutral breath-hold. All measurements were performed by a single operator with over 15 years of experience. The final UDFF value was calculated as the median of 10 valid measurements obtained under standardized acquisition conditions, IQR/Median < 30%.

### 2.6. Laboratory Assessment

Laboratory analyses were performed at the central laboratory of Ponderas Academic Hospital, accredited by the Romanian Accreditation Association (RENAR, Bucharest, Romania) in accordance with ISO 15189 standards. All procedures followed standardized protocols, and both internal and external quality control programs were applied to ensure the accuracy and reliability of the results.

The biochemical profile included liver enzymes, lipid parameters, fasting plasma glucose, fasting insulin, and insulin resistance assessment. The following variables were analyzed: alanine aminotransferase, aspartate aminotransferase, gamma-glutamyl transferase, triglycerides, HDL-cholesterol, fasting plasma glucose, fasting insulin, HOMA-IR, and HOMA-B.

### 2.7. Definition of Bariatric Success

Weight loss was expressed as percentage of excess weight loss (%EWL) and percentage of total weight loss (%TWL). The percentage of excess weight loss was calculated according to the following equation: %EWL = [(preoperative weight − postoperative weight)/(preoperative weight − ideal body weight)] × 100, where ideal body weight corresponded to a BMI of 25 kg/m^2^. Percentage of total weight loss was calculated as follows: %TWL = [(preoperative weight − postoperative weight)/preoperative weight] × 100 [8,9]. For the purposes of patient categorization and the clinical discordance analysis, bariatric success was defined as %EWL ≥ 50%. %TWL was retained and analyzed separately as a continuous postoperative weight-loss outcome.

### 2.8. Statistical Analysis

The statistical analysis was designed to evaluate whether UDFF and DXA-derived VAT provide complementary information to conventional bariatric follow-up parameters. The primary analysis assessed longitudinal changes in hepatic fat, visceral adiposity, anthropometric parameters, and metabolic profile between baseline and 6-month follow-up. Secondary analyses evaluated the association between UDFF reduction and changes in DXA-derived VAT, BMI, EWL, TWL, and selected biochemical parameters commonly used in bariatric follow-up.

Microsoft Excel (Microsoft Corporation, Redmond, WA, USA) was used to compose the database, and statistical analysis was conducted using IBM SPSS Statistics, version 26.0 (IBM Corp., Armonk, NY, USA).

Normality was assessed using the Shapiro–Wilk test. Paired comparisons between baseline and 6-month follow-up were performed using the paired Student’s *t*-test or the Wilcoxon signed-rank test, according to data distribution. Subgroup comparisons according to bariatric success were performed using the independent-samples *t*-test or the Mann–Whitney U test, as appropriate. The relationship between UDFF reduction and conventional bariatric follow-up parameters, including BMI reduction, EWL, TWL, liver enzymes, and selected metabolic parameters, was assessed using Pearson or Spearman correlation analysis, according to data distribution. A *p*-value < 0.05 was considered statistically significant.

Multivariable linear regression models were constructed to evaluate whether reductions in VAT and other selected body-composition parameters were independently associated with UDFF reduction after adjustment for BMI and HOMA-IR reduction. Candidate predictors were selected a priori based on clinical relevance, biological plausibility, and previous evidence linking visceral adiposity, regional fat distribution, overall adiposity, insulin resistance, and hepatic steatosis. Predictor selection was not based solely on statistical significance in univariable analyses. Given the sample size of 41 participants, each model was restricted to three predictors to reduce model instability and the risk of overfitting. Because VAT, android fat mass, and the android-to-gynoid ratio represent related body-composition measures, they were evaluated in separate models to limit multicollinearity. The multivariable regression analyses were considered exploratory and hypothesis-generating.

As an exploratory clinical discordance analysis, patients were categorized according to conventional bariatric success, defined as %EWL ≥ 50%, and residual hepatic steatosis at 6 months. %TWL was analyzed separately as a continuous weight-loss outcome and was not used for patient categorization. Residual hepatic steatosis was defined as UDFF ≥ 5%, according to the UDFF reference ranges, where values below 5% indicate the absence of steatosis. A sensitivity analysis was performed using UDFF ≥ 10%, corresponding to at least moderate residual steatosis. Group distributions were compared using Fisher’s exact test.

## 3. Results

### 3.1. Descriptive Analysis

A total of 41 patients were included in the study, with a mean age of 35.2 ± 11.3 years. The cohort consisted of a balanced sex distribution, with 41% male and 59% female patients.

At baseline, the cohort was characterized by severe obesity, increased visceral adiposity, insulin resistance, and quantitative evidence of hepatic steatosis. At 6 months after bariatric surgery, significant improvements were observed in anthropometric, body composition, metabolic, biochemical, and hepatic steatosis parameters. Mean EWL was 73.21 ± 25.66%, and mean TWL was 30.77 ± 7.66%. Overall, 35 patients achieved bariatric success, defined as EWL ≥ 50%, corresponding to 85.4% of the cohort. However, UDFF reduction did not differ significantly between patients with and without bariatric success.

**Figure 1:**
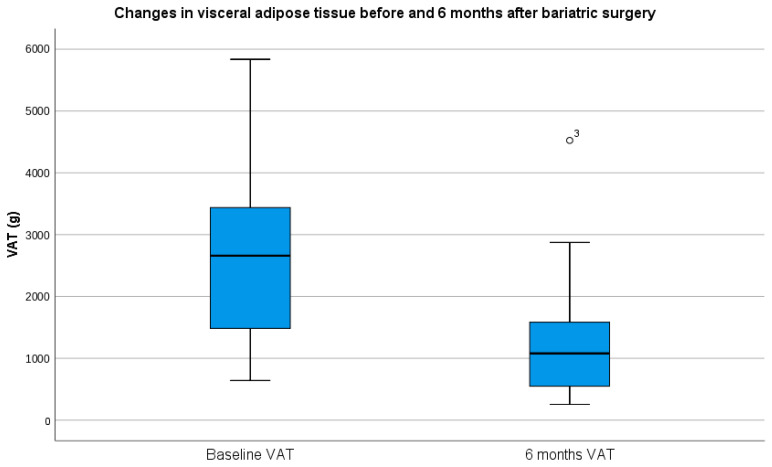
Boxplot changes in visceral adipose tissue (VAT) before and 6 months after bariatric surgery. The circle denotes an outlier (1.5–3 interquartile ranges from the box), and the adjacent number identifies the corresponding case.

**Figure 2:**
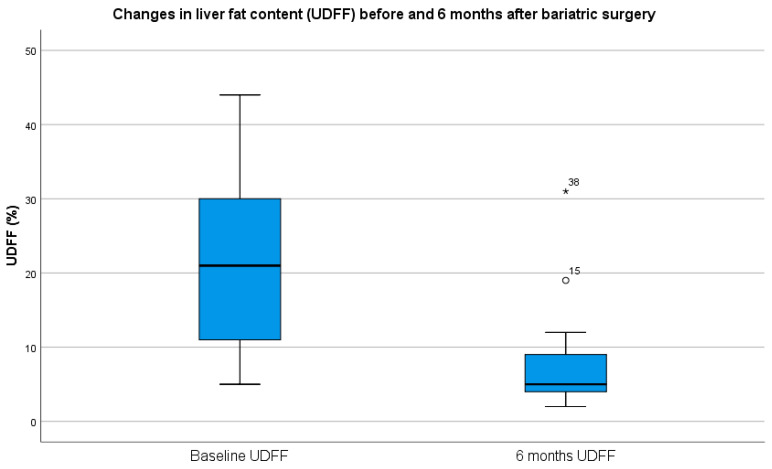
Boxplot changes in ultrasound-derived fat fraction (UDFF) before and 6 months after bariatric surgery. Circles denote outliers (1.5–3 interquartile ranges from the box), the asterisk denotes an extreme outlier (>3 interquartile ranges), and adjacent numbers identify the corresponding cases.

The complete paired comparisons between baseline and 6-month follow-up are summarized in Table 1. Changes in VAT and UDFF are illustrated in Figure 1 and Figure 2.

### 3.2. Association Between UDFF Reduction and Conventional Follow-Up Parameters

**Figure 3:**
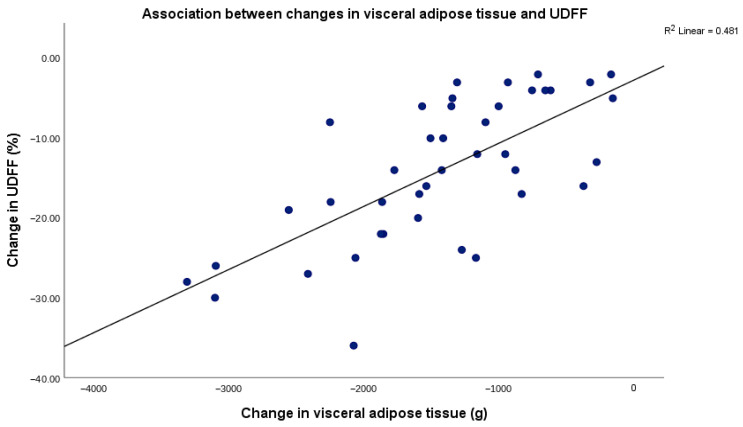
Scatter plot illustrating the association between changes in visceral adipose tissue (VAT) and changes in ultrasound-derived fat fraction (UDFF) following bariatric surgery.

Reduction in UDFF was strongly associated with reduction in DXA-derived VAT (ρ = 0.712, *p* < 0.001) (Figure 3) and android mass (ρ = 0.627, *p* < 0.001). A weaker association was observed between UDFF reduction and BMI reduction (ρ = 0.411, *p* = 0.008). In contrast, UDFF reduction was not significantly associated with EWL or TWL, irrespective of bariatric success. Whereas previous studies reported a strong correlation between reductions in UDFF and BMI (r = 0.75, *p* < 0.001), our findings suggest that changes in hepatic steatosis were more closely associated with reductions in DXA-derived visceral adipose tissue than with overall weight-loss metrics [10]. Correlation results are presented in Table 2.

### 3.3. Multivariable Regression Analysis

Multivariable linear regression indicated that DXA-derived VAT reduction was more closely associated with hepatic fat improvement than conventional weight-loss parameters and metabolic markers. After sensitivity analysis, none of the added metabolic and biochemical parameters remained independently associated with UDFF change after adjustment for VAT and BMI. Regression models are presented in Table 3.

### 3.4. Clinical Discordance Between Bariatric Success and Residual Hepatic Steatosis

Residual hepatic steatosis, defined as UDFF ≥ 5% at 6 months, was present in 29/41 patients (70.7%). Among patients who achieved bariatric success, 24/35 (68.6%) still had residual steatosis, compared with 5/6 (83.3%) of those without bariatric success (Fisher's exact test, *p* = 0.651). When a higher threshold corresponding to at least moderate steatosis was applied (UDFF ≥ 10%), residual moderate-or-severe steatosis was observed in 8/41 patients (19.5%). This was more frequent among patients without bariatric success than among those with bariatric success, 4/6 (66.7%) versus 4/35 (11.4%), *p* = 0.009. Resolution of steatosis, defined as UDFF < 5%, was found in 11/35 patients with bariatric success (31.4%) as compared with 1/6 patients without bariatric success (16.7%). The corresponding group distributions are presented in Table 4.

## 4. Discussion

The present study evaluated the combined role of UDFF and DXA-measured VAT for monitoring hepatic and body composition changes 6 months after bariatric surgery. The main findings were that both UDFF and VAT decreased significantly after surgery, and that VAT reduction showed the strongest association with UDFF reduction. This association was stronger than that observed for conventional bariatric follow-up parameters, including BMI reduction, EWL, and TWL. These findings suggest that UDFF and DXA-derived VAT provide complementary quantitative information beyond standard weight-loss outcomes. Our findings are similar to those of Algooneh et al., who demonstrated that the remission of hepatic steatosis was incomplete even among those with high EWL (>70%) [11,12]. In our study, although most patients achieved successful weight loss according to EWL criteria, residual hepatic steatosis was still observed in a substantial proportion of patients when UDFF was used as a liver-specific quantitative endpoint. Using the UDFF reference threshold of ≥5%, residual hepatic steatosis was present in 68.6% of patients who achieved bariatric success. When a higher threshold corresponding to at least moderate steatosis was applied, namely UDFF ≥ 10%, residual moderate-or-severe steatosis was still observed in 11.4% of patients with bariatric success, compared with 66.7% of those without bariatric success. The lack of a significant difference in residual steatosis between patients with and without conventional bariatric success is clinically relevant. This finding suggests that weight-loss success and hepatic steatosis resolution are related but not interchangeable outcomes. EWL reflects global bariatric response, whereas UDFF captures residual hepatic fat content. Therefore, some patients may achieve conventional weight-loss success while still presenting residual hepatic steatosis at 6 months. Alternatively, the UDFF ≥ 5% threshold may be highly sensitive for detecting minimal residual hepatic fat during early postoperative follow-up. In contrast, when a higher threshold corresponding to at least moderate steatosis was applied, residual steatosis was significantly more frequent among patients without bariatric success. These findings indicate that conventional weight-loss criteria do not fully exclude residual hepatic steatosis and support the added value of liver-specific monitoring after bariatric surgery [13]. The methodological distinction of our study lies in the use of DXA-derived VAT assessment, in contrast to previous studies that quantified VAT using abdominal ultrasound, a technique whose limitations in accurately assessing visceral adipose tissue distribution have been acknowledged [14].

The stronger association between VAT reduction and UDFF reduction is biologically plausible. Visceral adipose tissue is closely linked to hepatic lipid accumulation through increased free fatty acid flux to the liver, insulin resistance, low-grade inflammation, and altered adipokine signaling [15,16]. In the present cohort, VAT reduction was more closely related to hepatic fat improvement than global weight-loss metrics. This does not reduce the clinical value of BMI, EWL, or TWL, which remain essential measures of bariatric outcome. Rather, it suggests that these parameters capture only part of the postoperative metabolic response and may not fully reflect changes in intrahepatic fat content.

The role of UDFF in this context is particularly relevant because it provides a direct, quantitative, non-invasive estimate of hepatic fat content. Routine biochemical markers and serum-based steatosis indices may improve after bariatric surgery, but they do not directly quantify residual liver fat. In our study, metabolic and biochemical improvements were observed after surgery, and several of these parameters showed associations with UDFF reduction. However, their associations were weaker than that observed for VAT reduction. This supports the concept that UDFF serves as a liver-specific monitoring tool, while DXA-derived VAT reflects the visceral adiposity component of metabolic risk.

UDFF is a promising quantitative ultrasound-based method for hepatic fat assessment and has shown good agreement with MRI-PDFF in published validation studies [4,5]. However, validation data specifically focused on patients with severe obesity or bariatric surgery cohorts remain limited. In this population, increased body habitus and technically challenging acoustic conditions may influence ultrasound-based measurements. To address this issue, UDFF measurements in the present study were performed using the ACUSON Sequoia system equipped with the DAX-Deep Abdominal Transducer, which is specifically designed for deep abdominal imaging in patients with high body mass index [17]. Therefore, the present findings support the feasibility and clinical usefulness of UDFF for postoperative monitoring in patients with severe obesity, but do not represent a formal validation study against MRI-PDFF or histology. The combined UDFF–VAT (DXA) approach should be interpreted as a dual-compartment imaging framework, rather than as a mechanistic axis. UDFF reflects hepatic fat content, whereas DXA-derived VAT captures changes in visceral adiposity, a metabolically active fat compartment. Therefore, the combined assessment captures two biologically connected but distinct components of MASLD pathophysiology: hepatic steatosis and visceral adiposity. From a practical perspective, patients who achieve %EWL ≥ 50% but retain UDFF ≥ 10% at 6 months should not be considered complete hepatic responders solely on the basis of successful weight loss. Such patients may benefit from closer hepatological and metabolic follow-up, reinforcement of lifestyle measures, optimization of residual metabolic risk factors, and repeat liver-specific evaluation during follow-up. Our findings are consistent with previous studies showing that bariatric surgery reduces hepatic steatosis and improves metabolic dysfunction. However, the present study adds to existing evidence by integrating quantitative liver fat assessment with DXA-derived VAT measurement. Rather than relying only on weight-loss outcomes or biochemical markers, this dual-compartment monitoring approach may provide a more comprehensive characterization of postoperative improvement in patients with severe obesity and MASLD.

Several limitations should be acknowledged. First, this was a single-center study with a relatively small sample size, which limits generalizability and restricts the number of covariates that could be included in multivariable models. Consequently, the multivariable regression analyses should be interpreted as exploratory and hypothesis-generating rather than as definitive predictive models. Although the number of covariates included in the models was intentionally restricted, the sample size remains limited for robust multivariable modeling, and the possibility of overfitting and residual confounding cannot be excluded.

Second, the follow-up period was limited to 6 months, and longer-term studies are needed to determine whether UDFF and VAT changes persist over time.

Third, liver biopsy and MRI-PDFF were not available as reference standards for hepatic fat quantification. Although UDFF has shown good agreement with MRI-PDFF in published studies, data regarding the bariatric population remain limited. To address the technical challenges associated with ultrasound imaging in patients with severe obesity, UDFF measurements were performed using the ACUSON Sequoia system equipped with the DAX-Deep Abdominal Transducer, which is specifically designed for deep abdominal imaging in patients with high body mass index.

Fourth, the study included only patients undergoing laparoscopic sleeve gastrectomy, and the findings may not be directly applicable to other bariatric procedures.

Fifth, we did not systematically collect patient-level data on the prior pharmacological treatment of associated cardiometabolic factors, including each treatment class, treatment duration, and postoperative medication changes. However, no patient had diabetes mellitus or received antidiabetic therapy, reducing the likelihood that glucose-lowering medication confounded the observed changes in insulin resistance, body weight, and hepatic steatosis.

Finally, the present study should be interpreted within its intended scope, namely, the quantitative assessment of hepatic steatosis and visceral adiposity after sleeve gastrectomy. It was not designed to provide comprehensive MASLD staging. Fibrosis assessment was therefore beyond the scope of the study protocol. Although fibrosis is not required for the diagnosis of MASLD, it remains an important prognostic marker within the MASLD spectrum. Larger multicenter studies with longer follow-up are needed to confirm the generalizability and durability of these findings. Validation against MRI-PDFF and, where clinically indicated, histological assessment would further clarify the accuracy of UDFF for longitudinal hepatic fat monitoring in bariatric populations.

## 5. Conclusions

This prospective study showed that UDFF and DXA-derived VAT significantly decreased 6 months after bariatric surgery. VAT reduction was more strongly associated with UDFF reduction than conventional weight-loss outcomes, suggesting that visceral adiposity changes better reflect hepatic fat improvement than BMI, EWL, or TWL alone.

Residual hepatic steatosis was still observed in some patients despite conventional bariatric success, supporting the role of liver-specific quantitative monitoring during postoperative follow-up. The combined UDFF–VAT(DXA) assessment may provide complementary information by capturing both the hepatic fat response and visceral adiposity reduction. Larger studies with longer follow-up are needed to validate this dual-compartment imaging monitoring approach.

## Figures and Tables

**Table 1:** Baseline and 6-month cohort characteristics.

Variable	T0	T1	Δ = T0 − T1	*p*-Value
**Anthropometric and body composition**				
BMI, kg/m^2^	40.40 ± 6.43	28.33 ± 5.52	12.06 ± 3.49	<0.001
Waist circumference, cm	128.17 ± 12.70	101.78 ± 13.14	26.39 ± 7.65	<0.001
VAT, g	2721.8 ± 1411.9	1293.6 ± 916.4	1428.2 ± 791.3	<0.001
Android mass, kg	9.80 ± 2.17	5.87 ± 1.77	3.93 ± 1.20	<0.001
A/G ratio	1.22 ± 0.17	1.09 ± 0.22	0.13 ± 0.12	<0.001
**Weight-loss outcomes at 6 months**				
EWL, %	—	73.21 ± 25.66	—	—
TWL, %	—	30.77 ± 7.66	—	—
Bariatric success, *n* (%)	—	35/41 (85.4%)	—	—
**Insulin resistance and hepatic steatosis indices**				
HOMA-IR	7.34 ± 4.64	2.32 ± 1.79	5.02 ± 4.17	<0.001
UDFF, %	21.05 ± 10.79	7.05 ± 5.04	14.00 ± 9.02	<0.001
**Biochemical profile**				
AST, U/L	26.73 ± 7.81	22.38 ± 16.65	4.35 ± 15.61	<0.001
ALT, U/L	38.84 ± 17.14	23.94 ± 24.12	14.89 ± 28.71	<0.001
GGT, U/L	42.54 ± 39.99	19.29 ± 16.09	23.25 ± 28.09	<0.001
Fasting glucose, mg/dL	106.79 ± 28.85	84.86 ± 9.21	21.92 ± 26.41	<0.001
HbA1c, %	5.58 ± 0.86	5.03 ± 0.49	0.55 ± 0.53	<0.001
Insulin, µU/mL	28.84 ± 15.10	9.89 ± 5.13	18.95 ± 13.71	<0.001
HOMA-B, %	188.64 ± 99.24	161.83 ± 71.56	26.81 ± 97.71	0.087
Total cholesterol, mg/dL	203.07 ± 44.75	190.88 ± 44.54	12.20 ± 40.66	0.047
LDL cholesterol, mg/dL	127.82 ± 35.41	126.59 ± 41.10	1.22 ± 33.72	0.582
HDL cholesterol, mg/dL	45.67 ± 7.44	50.19 ± 6.28	4.52 ± 7.18	<0.001
Triglycerides, mg/dL	169.82 ± 105.15	92.97 ± 34.75	76.85 ± 95.72	<0.001
Albumin, g/L	46.31 ± 1.98	47.16 ± 2.52	0.86 ± 2.53	0.036

Note: Values are expressed as mean ± SD (standard deviation), unless otherwise specified. For longitudinal comparisons, change was calculated as T0 − T1. Therefore, positive values indicate postoperative reductions for variables expected to decrease after bariatric surgery. For variables that increased after surgery, including HDL cholesterol and albumin, the direction of change is indicated in the table. Bariatric success was defined as %EWL ≥ 50%. %TWL was evaluated separately as a continuous postoperative weight-loss outcome. BMI, body mass index; VAT, visceral adipose tissue; A/G ratio, android-to-gynoid ratio; EWL, excess weight loss; TWL, total weight loss; HOMA-IR, homeostatic model assessment of insulin resistance; UDFF, ultrasound-derived fat fraction; AST, aspartate aminotransferase; ALT, alanine aminotransferase; GGT, gamma-glutamyl transferase; LDL, low-density lipoprotein; HDL, high-density lipoprotein; HbA1c, glycated hemoglobin; HOMA-B, homeostatic model assessment of beta-cell function.

**Table 2:** Association between changes in UDFF and changes in anthropometric, body composition, metabolic, and biochemical parameters.

Parameter	Spearman ρ with ΔUDFF	*p*-Value
**Anthropometric and body composition**		
ΔVAT, g	0.712	<0.001
ΔAndroid mass, kg	0.627	<0.001
ΔBMI, kg/m^2^	0.411	0.008
ΔA/G ratio	−0.023	0.888
**Metabolic and biochemical parameters**		
ΔHOMA-IR	0.502	0.001
ΔFasting glucose, mg/dL	0.471	0.002
ΔInsulin, µU/mL	0.398	0.010
ΔHbA1c, %	0.274	0.083
HDL cholesterol increase, mg/dL	0.354	0.023
ΔGGT, U/L	0.326	0.037
**Weight-loss outcomes**		
EWL, %	−0.035	0.829
TWL, %	0.137	0.392

Note: Δ was defined as the change from baseline to 6 months after bariatric surgery, calculated as T0 − T1; positive Δ values indicate postoperative reduction. UDFF—ultrasound-derived fat fraction; VAT—visceral adipose tissue; BMI—body mass index; A/G ratio, android-to-gynoid ratio; HOMA-IR—homeostatic model assessment of insulin resistance; GGT—gamma-glutamyl transferase; EWL—excess weight loss; TWL—total weight loss; HbA1c, glycated hemoglobin; HDL, high-density lipoprotein.

**Table 3:** Multivariable linear regression models evaluating predictors of change in UDFF.

Predictor	Model 1 ΔVAT	Model 2 ΔAndroid Mass	Model 3 ΔA/G Ratio
R^2^	0.535	0.351	0.266
Adjusted R^2^	0.498	0.298	0.207
*p* model	<0.001	0.001	0.009
ΔVAT	β = 0.602, *p* < 0.001	—	—
ΔAndroid mass	—	β = 0.390, *p* = 0.032	—
ΔA/G ratio	—	—	β = −0.049, *p* = 0.731
ΔBMI	β = 0.216, *p* = 0.081	β = 0.062, *p* = 0.722	β = 0.279, *p* = 0.071
ΔHOMA-IR	β = 0.071, *p* = 0.596	β = 0.267, *p* = 0.075	β = 0.343, *p* = 0.028

Note: Δ values were calculated as T0 − T1; positive Δ values indicate postoperative reduction. Standardized β coefficients are reported. UDFF—ultrasound-derived fat fraction; VAT—visceral adipose tissue; BMI—body mass index; A/G ratio—android-to-gynoid ratio; HOMA-IR—homeostatic model assessment of insulin resistance.

**Table 4:** Clinical discordance between bariatric success and residual hepatic steatosis at 6 months.

Threshold	Bariatric Outcome	No Residual Steatosis	Residual Steatosis	*p*-Value
UDFF ≥ 5%	EWL ≥ 50%	11/35 (31.4%)	24/35 (68.6%)	0.651
UDFF ≥ 5%	EWL < 50%	1/6 (16.7%)	5/6 (83.3%)	
UDFF ≥ 10%	EWL ≥ 50%	31/35 (88.6%)	4/35 (11.4%)	0.009
UDFF ≥ 10%	EWL < 50%	2/6 (33.3%)	4/6 (66.7%)	

Note: Residual hepatic steatosis was defined as UDFF ≥ 5%. Residual moderate-or-severe steatosis was defined as UDFF ≥ 10%. Bariatric success was defined as EWL ≥ 50%. Percentages are calculated within each bariatric outcome group. *p*-values were calculated using Fisher’s exact test.

## Data Availability

The raw data supporting the conclusions of this article will be made available by the authors on request.
